# Effect Mechanism Research of Carbon Price Drivers in China—A Case Study of Shenzhen

**DOI:** 10.3390/ijerph191710876

**Published:** 2022-08-31

**Authors:** Jiongwen Chen, Jinsuo Zhang

**Affiliations:** 1 College of Energy Engineering, Xi’an University of Science and Technology, Xi’an 710054, China; 2 The People’s Government of Futian District, Shenzhen 518000, China; 3 School of Economics and Management, Yan’an University, Yan’an 716000, China

**Keywords:** carbon emission exchange, price drivers, effect mechanism, cointegration test, ridge regression

## Abstract

Revealing the effect mechanism of carbon price drivers is the basis to establish the pricing mechanism of carbon emission exchange, which also promotes the development of the carbon emission exchange market and can reduce the investment risk. Based on the previous research, the cointegration test, Granger causality test, and ridge regression estimate are used to analyze the effect mechanism between the domestic carbon price and its drivers. Johansen’s cointegration analysis reveals that there is a long-term equilibrium relationship between the domestic carbon price and energy price, industrial development level, climate change, and financial prosperity. Ridge regression estimates reveal that the international spot price of thermal coal in the ARA port and the spot price of Brent crude oil in Britain are negatively correlated with the domestic carbon price, while CER futures price is positively correlated with the domestic carbon price. There is a linkage between the international carbon price and the domestic carbon price. Since 2017, the domestic carbon price has been lower than the equilibrium value, and the value of carbon emission rights has been underestimated. With the continuous improvement of the domestic carbon market, the carbon price will rise and fluctuate around the equilibrium price in the future.

## 1. Introduction

Since the industrial revolution in Western countries, the increasing population, intensive agricultural activities, increased land use and deforestation, industrialization, rapid economic development, and fossil energy consumption have all contributed to the increase in greenhouse gas emissions. Elevated greenhouse gas emissions will cause global warming, which will seriously affect the sustainable development of our society. Therefore, reducing greenhouse gases (especially CO_2_) is the common interest and responsibility of people all over the world. It has been proven that the carbon emission trading mechanism is the most cost-effective method of emission reduction. China launched the national carbon market for the power industry in 2017, but it will take at least two years to achieve a national carbon market. The carbon market is a special market in which carbon price is affected by a variety of factors, such as international politics, macroeconomics, inter-country and domestic carbon allocation, technological progress, energy prices, and climate change. At present, China’s carbon market is still dominated by regional markets. A unified and perfect carbon emission trading market has not yet been established, which accounts for the fact that the carbon price drivers are still in dynamic evolution. Especially for China’s carbon market in its infancy, the carbon price drivers are also targeted [1]. However, only by analyzing the main carbon price drivers and their interaction mechanism effectively can a reasonable pricing mechanism be established. Compared with foreign carbon markets, the lack and strength of China’s carbon policy are the key reasons for the low carbon price. The main reasons for the low carbon price are that the carbon tax policy is not used, the carbon financial derivative mechanism is not applied in combination, and the policy setting in the total amount trading mechanism is relatively loose, the market mechanism is not perfect, and the proportion of forest carbon sequestration offset is not high. Therefore, by exploring the carbon price drivers and mechanism of a regional carbon price in China, this paper further reveals the relationship between the carbon price and market equilibrium price, which is not only conducive to the rational allocation of carbon emission quotas in China but also can promote the development of China’s carbon market, and even reduce investment risks and avoid the loss from carbon price fluctuations in the market as well.

## 2. Literature Review

Based on the market equilibrium theory, the carbon price mainly depends on the supply and demand of carbon emission exchange. Chen Xiaohong and Wang Rongyun, [2] by taking the EU emission trading system as the research object, found that market supply and demand were important factors affecting carbon price, and that quota supply affected by policies and systems is the most important factor affecting the transaction price. Zhongjue Yu and Yong Geng [3] used the computable general equilibrium model (CGE) to evaluate the impacts of the macro-economy, carbon trading market, and participating sectors on carbon emissions in 2030. The results show that the carbon price will be USD 161.2 per ton and USD 147.2 per ton in these two representative cases, and the price will be USD 161.2 per ton and USD 147.2 per ton under certain emission reduction targets. The initial allocation subsidy has a significant impact on the trading behavior of the carbon trading market. Tang and Shen [4] used capital asset pricing models to analyze the market risks in the European Union’s Carbon Emission Trading System (EU ETS) and clean development mechanism (CDM). Zipf analysis is used to analyze the volatility of carbon prices in the two markets with different expected returns. The results show that the EU carbon price is more unstable and risky than that of the CDM market under high expected returns. Creti and Jouvet [5] studied whether there were differences in the determinants of carbon prices in the two stages of the European Union Emissions Trading Scheme (EU ETS). It was found that the carbon price drivers of the second stage of carbon prices were significantly different from those in the first stage. The impact of oil price, equity price index, and natural gas on coal conversion cost in the second stage of a carbon price is obvious. While a cointegrating relationship exists for both phases of the EU ETS, the nature of this equilibrium relationship is different across the two subperiods, with an increasing role of fundamentals in Phase II. Jinpeng Li and bei Xu et al. [6] take Beijing as an example, using the system dynamics analysis model and identified the key factors and evolution path of carbon emission mechanism from three aspects of society, including energy, economy, and environment. The results show that the carbon emission trading mechanism is different under different scenarios. ChevallierJ’s research challenged the market observers’ viewpoint that carbon futures prices are immediately correlated with changes in the macroeconomic environment, and suggest that the carbon market is only remotely connected to macroeconomic variables. The economic logic behind these results may be related to the fuel-switching behavior of power producers in primarily influencing carbon futures price changes [7]. Relevant studies have further confirmed that there is a long-term stable relationship between international carbon futures prices and domestic carbon prices, showing an obvious one-way causality [8], which helps to predict carbon prices [9].

In terms of specific influencing factors, the carbon price is influenced by multiple factors, such as government policy, internal market mechanism, and external market environment. Under the internal market mechanism, the interaction between energy price and carbon emission trading price is interactive, among which oil price has the greatest impact on carbon price; under the external market environment, the quarterly GDP growth rate, temperature, and precipitation of regional development have different degrees of impact on carbon price [10]. Besides supply and demand, carbon emissions’ exchange price is also affected by industrial development level, traditional energy price, financial market development, and climate change [11,12,13], and the change in carbon emissions trading price will affect the development of the macroeconomy. In terms of the impact mechanism, since fossil energy consumption is the main source of carbon emissions, energy market changes are key aspects of the price formation mechanism in the international carbon trading market [14].

To explain the complex influence mechanism of the carbon market, the commonly used methods are the system dynamics method [6], structural equation model [12], VAR model [14], and other methods [15,16,17]. These methods still have shortcomings in revealing long-term equilibrium relations and eliminating multiple collinearities. The above research is mainly based on foreign carbon market trading data, and the conclusions are common. However, there is still a lack of in-depth study on the factors affecting the price of carbon emissions trading at the pilot stage in China, and the mechanism between the price drivers of domestic carbon emissions trading has not been fully revealed. Using data from four pilot markets in Beijing, Guangdong, Hubei, and Shenzhen from 2014 to 2016, Fan and Todorova found that different regional markets have different influence factors on carbon price [18]. This shows that compared with the European carbon market, China’s carbon market is at an early stage of development, and the efficiency of the carbon market is relatively low. The relationship between the carbon price and its influencing factors is a contingency and dynamic change [19]. Therefore, when the domestic carbon emission trading market develops to a new stage, a comprehensive approach should be adopted to analyze the new form of interaction between the carbon price drivers of the carbon market.

Our contributions are threefold. First, ours is the first study to apply the ridge regression method to identify the factors that drive the price of carbon emission rights and identify the carbon price drivers. Second, it reveals the long-term equilibrium trend among the carbon price drivers. Third, our empirical results, to some extent, indicate that there is a linkage between the international carbon price and domestic carbon price.

## 3. Identification of Carbon Price Drivers

### 3.1. Methodology

In this study, ridge regression is used to simulate the relevant scenarios. The implementation path of ridge regression to eliminate the influence of multi-collinearity is to introduce the nonnegative factor to the main diagonal of the independent variable matrix. The introduction of the nonnegative factor will inevitably have a negative impact on the overall goodness of fit of the model to a certain extent, but the introduction of the factor will also make the empirical results more stable and effective.

### 3.2. Carbon Price Drivers

**Figure 1:**
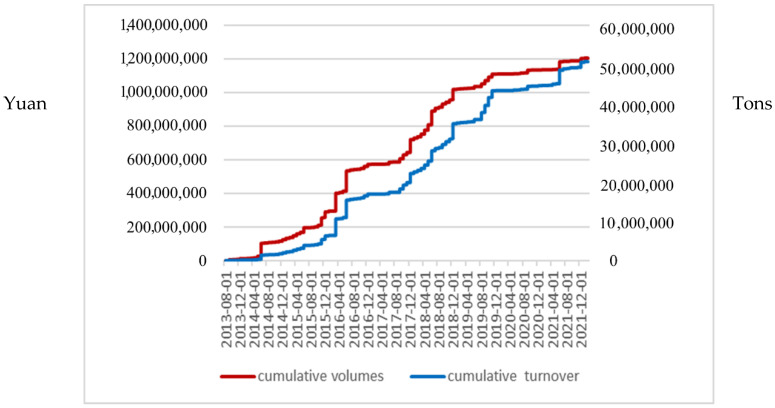
Average trading volume and price of Shenzhen carbon emissions market.

Since 2005, China has been the world’s largest energy consumer and carbon emitter, with increasing carbon emissions year by year. In China, the use of market mechanisms to control greenhouse gas emissions started with the clean development mechanism under the Kyoto protocol. China’s current carbon trading market is still in the early stage of construction. Starting from the CDM project in the Kyoto Protocol, it is divided into the following three stages: the clean development mechanism stage (2002–2011), the pilot trading stage (2011–2017), and the national trading stage (2017-today). The local quota spot and China Certified Emission Reduction (CER) spot are two trading varieties in China’s carbon trading market during the pilot period, with local quota as the leading one. Since 2011, seven carbon trading pilots have been launched in Beijing, Tianjin, Shanghai, Chongqing, Guangdong, Hubei, and Shenzhen to explore the establishment of a carbon trading mechanism. Among them, Shenzhen Carbon Emission Rights Exchange is the first carbon emission rights exchange in China, and also the most active one in China. The average trading volume and price in the Shenzhen carbon market are shown in Figure 1.

According to the daily closing price data of seven carbon trading pilots from 2013 to 2021, the carbon prices of seven carbon markets showed a downward trend in general from 2013 to 2021. In 2016, the average transaction prices of seven carbon markets gradually stabilized at about 25 yuan/ton [20]. Although the carbon price fluctuation in each market presents different persistent and asymmetric characteristics [21], it has a convergence in the range and trend [12,16,20]. Based on the fact that the Shenzhen Carbon Emission Exchange is the earliest pilot for carbon emission exchange in China, one can easily analyze the mechanism between driving factors. Taking the consistency of the data period into account, this paper chooses the daily average carbon price (SZA) of the Shenzhen Carbon Emission Exchange from September 2013 to March 2021 for this research. The carbon price data are from Shenzhen Carbon Exchange and Wind database, and the carbon price of Shenzhen is abbreviated as Carbon.

The influence mechanism of the traditional supply–demand relationship on the price is heterogeneous in China’s carbon market. For example, the influence of information at the level of the supply exchange on carbon price is significant, while the influence of information at the level of the government is not significant. This shows that the price drivers of the carbon emission rights market in China are different from those of the mature carbon emission rights markets abroad, and even contradict the theory [22]. This is also related to the development stage of China’s carbon market and the imperfect market mechanism, which is also in the “rational” process. Based on the pricing mechanism of carbon emission rights and the existing research literature, in this paper, carbon price drivers are revealed, including politics and system, climate change, carbon quota, macro-economy, energy price, and international carbon price, as shown in Table 1.

### 3.3. Identification of Key Drivers

The stage of China’s carbon market construction is an important scenario to analyze the drivers of China’s carbon emissions exchange price. At present, China’s carbon trading market is still in the pilot stage of decentralization, and the construction of a unified national carbon market has not yet been fully completed. Generally speaking, it is characterized by high policy sensitivity, high quota concentration, and poor availability of information data. According to the analysis of Table 1, there are still some deficiencies in the study of carbon price drivers of carbon emissions trading. First, the energy consumption structure of Europe or the United States is different from that of China, which makes foreign countries focus mainly on oil and natural gas prices when considering the energy price. However, China’s energy consumption is dominated by coal, which makes China distinct from Europe or the United States in the specific composition of carbon price drivers of energy price in carbon emission exchange price. Second, the financial market, especially the domestic financial market, will play an increasingly important role in the carbon price. For example, the fluctuation of carbon spot prices can reduce the volume of financial options trading and achieve the emission reduction target [36,37]. However, the research in this field has not achieved good results either at home or abroad. Third is the transformation and development of China’s industry. According to statistics, the total value added by China’s industry reached 28 trillion yuan, accounting for 33.9% of GDP in 2017, and China’s industry is entering the middle and late stages of industrialization as a whole. The impact of China’s industrial development on the carbon price should be studied as soon as possible. Fourth, when studying the impact of climate change on a carbon prices, we should pay more attention to air quality as well as temperature. Therefore, based on the existing research, this paper will focus on the price drivers of carbon emissions trading that have been neglected or not studied in-depth in previous studies, such as coal price, financial market, industrial development level, and air quality.

## 4. Effect Mechanism of Driving Factors

### 4.1. Indicators

(1) Carbon Price

There are obvious interactions in the international carbon market. For example, the certified emission reduction future price of the European Climate Exchange will influence the domestic regional carbon price. It is necessary to consider the relationship between different carbon prices in the study. To reveal the latest form of this relationship by using the latest data, this paper selects the Certified Emission Reductions futures price as the driving factor, abbreviated as “CER”.

(2) Energy Prices

China is the largest energy consumer, accounting for 23% of the world’s energy consumption. Energy prices will affect energy consumption and affect carbon dioxide emissions. Rising energy prices will lead to a decline in energy demand, especially for high-carbon energy, which will reduce the demand for carbon emission quotas, and the carbon price will fall. Based on the fact that the main energy consumption in China is coal, oil, and other traditional high-carbon energy sources, the spot price of thermal coal in the ARA Port in Europe, spot price of Brent crude oil in Britain, coal price in Qinhuangdao Port (Q5500) and the spot price of crude oil in the circum-Pacific Ocean (China’s victory) are selected as explanations to measure domestic and foreign energy prices. Variables are abbreviated as “ARA-coal”, “Brent”, “coal” and “crude”, respectively.

(3) Financial Market Prosperity

According to the previous analysis, the development of the financial market will become an important indicator of the activity of the carbon financial market. The development of the financial market will lead to the activity of the carbon trading market, that is, the prosperity of the financial market and the activity of the carbon market will change in the same direction. In this paper, the Shenwan 300 index is used as an index to measure the prosperity of the domestic financial market, which reflects the development of the domestic financial market and is abbreviated as “index”.

(4) Domestic Industrial Development Level

From the perspective of supply and demand, the carbon price is affected by the total supply and demand of carbon emission quotas. The quota system is the main trading mode in the domestic carbon emission exchange market at present, and supply has little influence on the carbon price. The demand for carbon emission rights of enterprises can be expressed by the difference between actual emissions and emission allocation. The demand for carbon emission rights of enterprises is closely related to macroeconomic development and the stage of industrialization. This paper uses the CSI index from September 2013 to August 2021 to represent the level of industrial development in China. The data are from Sina Finance and Economics, which is abbreviated as “indus”.

(5) Climate Change

Climate change is an important indicator that affects the fluctuation of carbon prices. Low or high temperatures will lead to a sharp increase in energy demand and greenhouse gas emissions, which will lead to an increase in demand for carbon emission rights. There is a linkage between air quality and carbon emissions. In this study, the daily maximum temperature in Shenzhen was selected as the driving factor; for a more precise study, we calculate the average temperature in Shenzhen ranging from 2013 to 2021. If the daily average maximum temperature in Shenzhen is higher than the average temperature in comparison to 2013, it is recorded as 1 (the weather is hotter), otherwise, it is recorded as 0 (the weather is colder). The average temperature and the air quality in Shenzhen are abbreviated as “temp” and “air”.

### 4.2. Effect Mechanism

**Figure 2:**
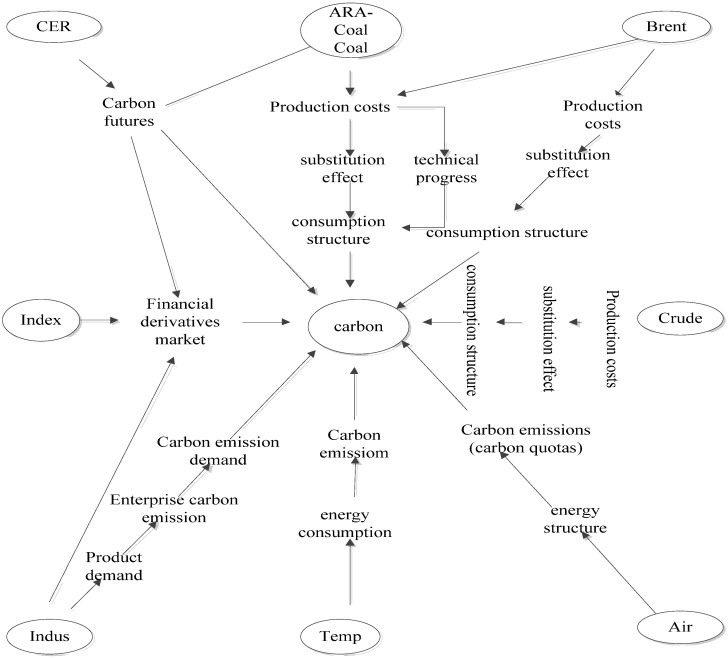
Effect mechanism of carbon price drivers.

In terms of specific driving factors, this paper chooses CER futures price, ARA-coal price, Brent crude oil spot price, Qinhuangdao port thermal coal (Q5500) price, Pacific crude oil spot price (China’s victory) daily trading price, Shenwan 300 index, CSI industry index, mean temperature and air quality in Shenzhen, which includes nine price carbon price drivers of the carbon price. Based on theoretical analysis, the above factors should have the following effect mechanisms: ① The price discovery function of the CER spot market is weak. The futures price is the Granger cause of the spot market. There is a long-term stable equilibrium relationship between the spot market and the futures market. CER price acts on the domestic carbon spot market through the futures market. ② ARA-coal, Brent, coal and crude follow the path of “enterprise production cost substitution effect-consumption structure-demand for carbon emission rights-carbon price”. ③ When encountering sudden climate change, such as cold winter or typhoon rainstorm, the demand for energy increases greatly, which leads to the increase in carbon emissions, and then the demand for carbon emission rights increases greatly, which leads to a sharp rise in the carbon price. ④ Requirements for air quality will prompt the government to enact laws to adjust the energy structure, and will also force enterprises to join the emission reduction program. ⑤ The Shenwan 300 Index will affect the price of financial derivatives, thus affecting the carbon market. ⑥ The CSI index affects carbon prices, along with the “product demand-Corporate carbon emission and carbon emission demand”. The above mechanism is shown in Figure 2.

Interventional studies involving animals or humans, and other studies that require ethical approval, must list the authority that provided approval and the corresponding ethical approval code.

### 4.3. Mechanism Analytic Model

**Figure 3:**
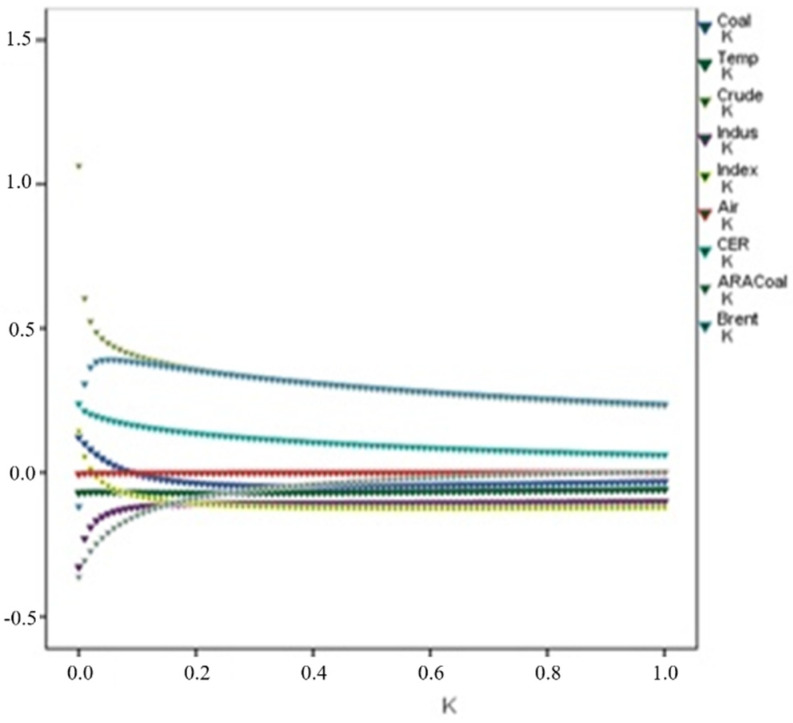
Ridge regression results.

According to Figure 3, the following two problems need to be solved: first, whether the theoretical relationship can be tested by actual data; second, the correlation between carbon price drivers needs to be further analyzed. Therefore, the unit root test of variables (carbon, CER, ARA-coal, Brent, coal, crude, indus, index, air) is carried out by using an econometric model. Since the virtual variables will not affect the co-integration relationship between the domestic carbon price and its drivers, the co-integration test and the grand test are applied to the 10 variables selected in this paper. Considering that there may be multiple collinearities in the selected variables in this paper, we use the ridge regression method to estimate, and a multiple linear regression model between the domestic carbon price and its drivers is constructed.

Carbon_t_ = *α*_0_ + *α*_1_CER_t_ + *α*_2_ARA-Coal_t_ + *α*_3_Brent_t_ + *α*_4_Coal_t_
+ *α*_5_Crude_t_ + *α*_6_Indus_t_ + *α*_7_Index_t_+ *α*_8_Air_t_ + *α*_9_Temp_t_ + *ε* (1)

where t represents the date of the study, $\alpha_{0}$ represents the intercept, $\alpha_{1}\sim \alpha_{9}$ represents the regression coefficients, and *ε* represents the residuals.

## 5. Empirical Analysis

### 5.1. Data Sources

For the accuracy of our research, first, the data are processed and the consistency of data research intervals is ensured. The specific variables and data sources with consistency processing are shown in Table 2.

### 5.2. Descriptive Statistics

The sample size of each variable is 1406, and the descriptive statistics of variables are shown in Table 3.

### 5.3. Johansen Cointegration Test

The Johansen cointegration test model is used to verify whether there is a long-term equilibrium relationship between the variables of the above multiple regression model. The cointegration test result is shown in Table 4.

From Table 4, we can observe that at a 1% significance level, the null hypothesis is refused. The results show that there are three co-integration relationships between the domestic carbon price and its drivers. The results provide more reliable proof of the long-term equilibrium and stable relationship between the domestic carbon price and its drivers. In long run, the relationship between variables selected in this paper maintains a long-term equilibrium.

### 5.4. Granger Causality Test

The cointegration test has shown that there is a long-term equilibrium trend among the variables. To further study the relationship between the domestic carbon price and its drivers, 10 variables were tested by the Granger causality test. According to the SC minimum principle, the lag period was finally selected as the fourth order. The result of the Granger causality test is shown in Table 5.

The Granger causality test shows that carbon price is affected by price drivers both in the domestic market and international market. There is an obvious Granger causality between energy prices (such as crude oil price and coal price) and domestic carbon prices. The temperature has no significant impact on the domestic carbon price, but the domestic financial market prosperity and carbon price show Granger causality. This means that there is a linkage effect between the domestic carbon market and the energy market, and there is an interaction between the carbon price and the energy price, which, to some extent, strengthens the impact of the domestic carbon emission exchange market on the domestic economy.

### 5.5. Ridge Regression Estimation

The cointegration analysis verifies that there is a long-term stable relationship between the domestic carbon price and energy price, financial market prosperity, industrial development level, and climate change. To study the equilibrium relationship between the domestic carbon price and its drivers, it is necessary to obtain the correlation coefficient among the variables, and the correlation test is shown in Table 6. From the test results, it can be observed that the correlation between some index variables is high, and significant at a 1% significance level. Firstly, ordinary least squares estimates are made for multivariate linear regression models, and the results of estimation coefficients are shown in Table 7.

From Table 7, it can be observed that the estimated coefficients of some variables have not passed the significance test, the F statistics and R^2^ are very high, and the VIF value (expansion factor) is also high, indicating that there is a correlation between the variables. To further verify whether there is serious multicollinearity between the variables, a multicollinearity diagnosis is made for the variables. There is serious multicollinearity between the variables, and the reliability of the regression coefficient obtained by the ordinary least square method is low. The influence of multicollinearity must be eliminated to avoid the phenomenon of false regression.

To make our research more accurate and eliminate the impact of multiple collinearities of variables, we use the ridge regression method to determine the correlation between the domestic carbon price and its drivers. The ridge trace is obtained by running the grammar program (Figure 3). According to the ridge regression result, k = 0.1 is obtained. When k = 0.1, the ridge regression estimation results are shown in Table 8. From Table 8, we can observe that the VIF value is significantly reduced and the multicollinearity is eliminated.

## 6. Results and Discussion

### 6.1. Empirical Results

The Johansen’s co-integration test results show that there are at least three co-integration relationships between the domestic carbon price and its drivers, indicating a long-term equilibrium. The Granger causality test shows that carbon price is affected by price drivers both in the domestic market and international market. The Ridge regression analysis is reflected in the following aspects.

The air temperature coefficient is not significant at 10%, which is inconsistent with the conclusions of Ma Huimin et al. [11]. The reasons may include the following: (1) in our research, we take Shenzhen Carbon Emission Exchange as an example, and the average temperature of Shenzhen is chosen as the research sample. Shenzhen is a transitional subtropical to a tropical oceanic climate, with an annual average temperature of 22.5 °C. The range of change is much smaller than that in the northern region, so the impact on carbon price is not obvious. (2) Previous studies have shown that temperature will affect carbon price. However, the temperature change ca not directly affect the price of carbon emission rights, and the transmission process is more complex. Therefore, this study shows that the impact of temperature on the price of domestic carbon price is not significant.

The coefficients of ARA-coal and Brent are significantly negative, and the CER coefficient is significantly positive. The domestic carbon price increased by 17.9% during the same period when the international CER price increased by 1%. For each 1% increase in the ARA-coal spot price and Brent spot price, the domestic carbon price fell by 0.2% and 0.26%, respectively, in the same period. This shows that the domestic carbon market is greatly influenced by the international energy market, and there is a linkage between the international energy price and the domestic carbon price.

The coefficients of coal price and crude oil spot price (China’s victory) are significantly negative, and the indus coefficient is significantly positive; the index coefficient is positive, but not significant. Energy prices, such as steam coal prices and crude oil prices, harm domestic carbon prices. If domestic oil prices rise by 1%, the carbon emissions price fall by 0.26%. The level of industrial development has a positive impact on the domestic carbon price. However, the impact of financial market prosperity on the carbon market is not significant, which is also inconsistent with the previous research. The reason may be that the previous research has not taken into account the multiple collinearities among the price drivers, and the domestic carbon futures market has not yet been established, which leads to the failure to establish the transmission mechanism of the financial market to the carbon emission exchange market. In addition, the impact of the financial market on carbon price is weak.

### 6.2. Equilibrium Carbon Price

Through the Johansen cointegration test, Granger causality, and ridge regression analysis, the regression equation of the equilibrium domestic carbon price drivers is obtained as follows:

Carbon = 17.90157308 ∗ CER − 0.00115989 ∗ Coal − 0.26584276 ∗ Crude − 0.03278454 ∗ Air − 0.20011181 ∗ ARA-Coal − 0.2618561 ∗ Brent + 0.00304198 ∗ Index + 0.00235551 ∗Indus − 2.6645335 ∗ Temp + 34.16795176 = 17.90157308 ∗ C2 − 0.00115989 ∗ F2 − 0.26584276 ∗ I2 − 0.03278454 ∗ H2-0.20011181 ∗ D2 − 0.2618561 ∗ E2 + 0.00304198 ∗ K2+0.00235551 ∗ J2 − 2.6645335 ∗ G2

**Figure 4:**
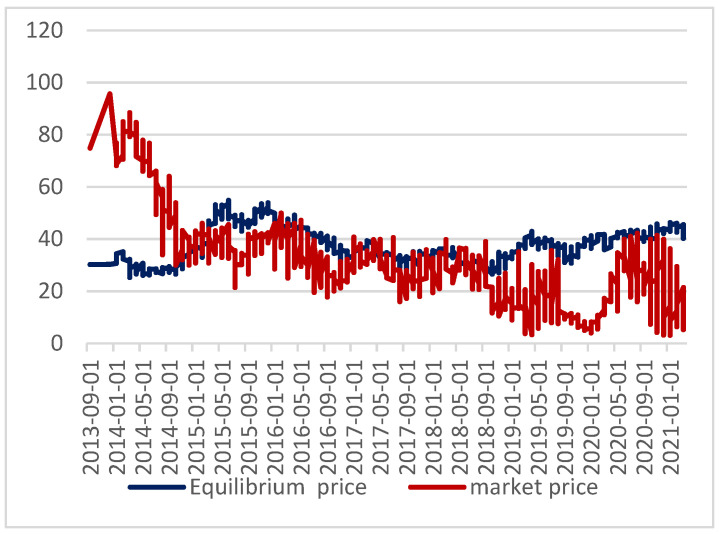
Equilibrium price and market price.

**Figure 5:**
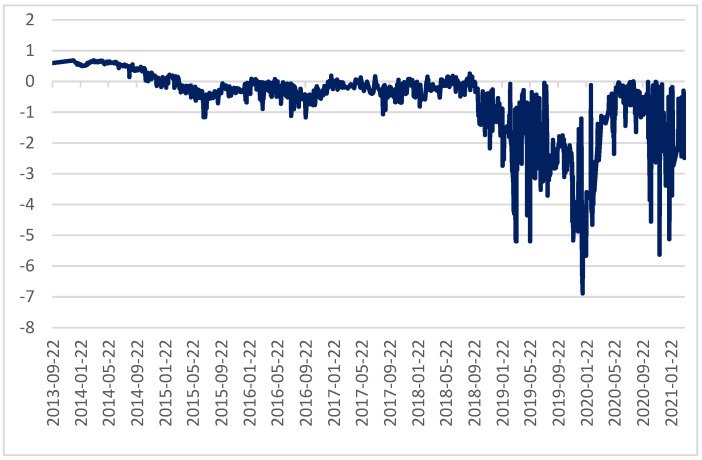
Relative error of equilibrium price and market price.

To study the relationship between the domestic carbon price and equilibrium price, this paper calculates the equilibrium price and error, reveals the formation mechanism of the domestic carbon price, and verifies the accuracy of the estimation of this model. The equilibrium price and market price are shown in Figure 4, and the price deviation is shown in Figure 5. In January 2021, the price deviation was large, with the relative error reaching 7%. In the rest of the period, the relative error was within 7%. When the carbon price is higher than the equilibrium price, the value of carbon emission rights is overestimated; when the carbon price is lower than the equilibrium price, the value of carbon emission rights is underestimated. Combining Figure 4 and Figure 5, it can be observed that the underestimation and overestimation of carbon emission rights’ value alternate in the whole study period. In 2013, the carbon price was higher than the equilibrium price, probably because the domestic carbon emissions trading market had just started in 2013, and market participants were more enthusiastic about new things. From May 2014 to November 2014, the value of domestic carbon emissions rights was undervalued and underestimated. With the entry into force of the Kyoto Protocol and the mandatory emission reduction requirements put forward by developed countries to developing countries, the demand for carbon emission rights increased in 2015 and the value of carbon emissions was overvalued.

From the whole research period, the underestimation period usually occurs in summer, and overestimation occurs mostly in winter. This may be due to the increasing consumption of coal, oil, and other traditional energy resources in winter, which leads to the increasing demand for carbon emission rights, driving up the price of carbon emissions trading. Industrial production activities are active, the government actively responds to the emission reduction policies put forward by developed countries, assumes the responsibility for emission reduction, and the carbon emission trading market develops rapidly, which, to a certain extent, has led to the development of the domestic macro-economy.

### 6.3. Analysis of Driving Path System

Under a market economy, the carbon price is mainly determined by the supply and demand of carbon emissions. In the short term, the supply of carbon emission is inelastic, so the carbon price derivers mainly depend on the demand side.

**Figure 6:**
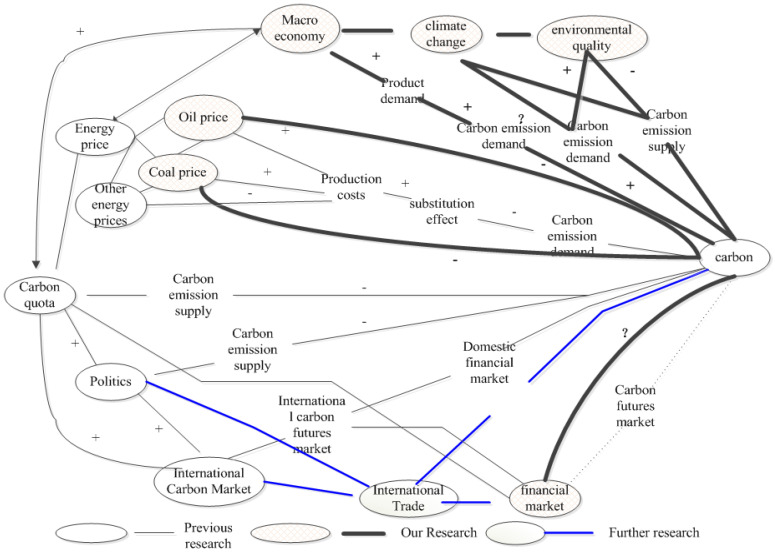
Effect path of carbon price drivers.

The research shows that the carbon price derivers are multi-dimensional, multi-level, and multi-path. For example, under the overall price derivers of energy price, the impact of coal price, oil price, natural gas price, electricity price, and new energy price on carbon emission is quite different. The impact of coal price on carbon price can be subdivided into the following two situations: short term, without considering technological progress and long term, with technological progress. The interaction between factors may be more complex. For example, the relationship between energy prices and macroeconomic development is difficult to simply define as a causal relationship. The change in energy price differences will be the key factor that affects the demand for carbon emissions. In particular, the rising price of low-carbon energy will force enterprises to choose low-cost, high-carbon energy for production. In the case of lower marginal production costs, enterprises will reduce the demand for carbon emissions. In theory, the temperature change will lead to an increase in energy demand and carbon price, but the empirical results show that the relationship between temperature change and carbon price is not significant in the short term. It is worth mentioning that the international trade structure will be an important factor that affects the carbon price [38], and the interaction between the two needs further study [39]. Combining the research in this paper and the previous research, the path of price drivers in the carbon emission exchange can be described in Figure 6.

## 7. Conclusions and Recommendations

### 7.1. Conclusions

Based on the daily trading price data of the Shenzhen carbon emission rights market from 2013 to 2021, the mechanism of price drivers in domestic regional carbon emission exchange is studied by using the co-integration test, Granger causality test, and ridge regression estimation. The conclusions are as follows: The spot price of thermal coal in the ARA port and spot price of Brent crude oil in Britain are negatively correlated with the domestic carbon price, while CER futures price is positively correlated with the domestic carbon price. There is a linkage between the international carbon price and domestic carbon price, and there is a long-term equilibrium relationship between the domestic carbon price and energy price, industrial development level, climate change, and other factors. Since 2017, the domestic carbon price has been lower than the equilibrium value, and the value of carbon emission rights has been underestimated. With the continuous improvement of the domestic carbon market, the carbon price will rise and fluctuate around the equilibrium price in the future.

### 7.2. Policy Recommendations

With the rapid development of the domestic economy and the strengthening of internationalization, the domestic carbon market will stay in line with the international carbon market. China is a large country with significant carbon emissions. In 2016, domestic carbon emissions accounted for 1/3 of the total carbon emissions in the world. For the sustainable development of the global economy, China takes the initiative to undertake emission reduction responsibilities, and the development of a domestic carbon emission trading market will have a greater impact on China’s future energy and economic development.

Firstly, the futures price of internationally certified emission reduction is positively correlated with the domestic carbon price. China should speed up the construction of the carbon market and futures market, improve the internationalization level of the carbon market, and realize the docking of the domestic carbon market and international market. One can learn from the developed countries when constructing a carbon futures market. Therefore, the market participants can change their positions in two markets to avoid the loss caused by the impairment of carbon assets and maximize profits.

Secondly, domestic traditional energy prices have a significant impact on the carbon emission price. Domestic enterprises should weigh up the difference between the cost of carbon emission rights and the cost of energy substitution, and find the best way to save energy and reduce emissions. Coal accounts for 29.2% of global primary energy consumption, and the total domestic coal consumption accounts for 64% of China’s energy consumption [40]. Coal has always been in the leading position in China’s energy consumption structure in the past few decades. The government strengthens the monitoring of traditional energy consumption, and actively advocates for enterprises to achieve industrial transformation and upgrading.

Third, upgrading the industrial structure is particularly important for reducing carbon emissions from energy consumption. The level and structure of international trade will be another important carbon price driver. It is suggested that further reform is deepened in all respects to force the upgrading of domestic industries, promote the economy to enter a stage of high-quality development, promote energy conservation, and enhance the pricing authority in the carbon market.

## Figures and Tables

**Table 1:** Carbon price drivers for the price of carbon emission.

Driving Factors	Main Empirical Literature	The Specific Role of Driving Factors
Politics and system	Alberola and Chevallier [13], Chen Xiaohong and Wang Zhengyun, Wang Zhonghua and Hu Biao, Zhao Lixiang and Hu Can [2,10,12]	Global governance responsibilities and political and legal systems can affect climate change response policies and then affect carbon prices, but the impact on carbon price is long term and mutative (once a political act and law is implemented, carbon prices will jump, and when the market absorbs its information, the impact on carbon price volatility is becoming weaker).
Climate factors	Alberola and Chevallier [13], Boeters [23], Michael Jakob [24]	Climate factors play an important role in the change in carbon prices. Extreme weather changes, whether overcooled or overheated, will lead to increased use of coal, natural gas, and other resources, resulting in carbon emissions, thereby raising carbon prices.
Carbon quota	ShihongZenga [25], Zhang Y J and Wang A D [26]	The carbon quota is an important supply factor; unreasonable allocation of carbon quota will also reduce the activity of the carbon market; intertemporal reserve will also affect carbon prices.
Macroeconomy	Yongda He and Boqiang Lin [27], Qiang Liu and Xiaoqi Zheng [28]	When the macro-economy is in the upstream cycle, especially the development of the industrial economy, the demand for products in the market increases, and the demand for carbon emission quotas in enterprises increases, so the carbon price rises; on the contrary, the carbon price falls.
Energy price	Zhang Yuejun and Wei Yiming, Qiang Ji and Dayong Zhang [29], Lutao Zhao and Lingyun He [30], Xin Zhao and Eng Han [31]	Energy price is the main influencing factor of carbon emission price, especially Brent oil price in the European market and coal price in the Chinese market.
International carbon future price	Zou Shaohui and Zhang Tian [8], Zhang Yuejun and Wei Yiming [15], Sun Chun [32]	There is a long-term stable relationship between international carbon futures price and domestic carbon price, showing an obvious one-way causality, and the domestic carbon market is relatively fragile and in a passive position.
International carbon price	Liu Ling and Zhang Rongrong [33], Guochang Fang and Lixin Tian [34], Renner M [35]	As the largest supplier of carbon emission rights in the world, to a great extent, China’s carbon emission exchange depends on the major international carbon markets, in which the EU carbon trading market is the most important carbon market; the fluctuation and decline in the international carbon price affect the trend of China’s carbon price to a certain extent and cause the same direction change in China’s carbon price. There is a long-term equilibrium relationship between EUA and CCER, but CCER is in a passive position.

**Table 2:** Data sources and descriptions.

Variable Description	Indicator Description	Variable Abbreviation	Data Sources	Unit
Carbon price drivers in the international market	Futures settlement price (continuous): certified emission reduction	CER	European Climate Exchange	Euro/ton carbon dioxide equivalent
	Thermal coal price of ARA port in Europe	ARA-coal	WIND database	Dollar/ton
	The spot price of Brent oil in England	Brent	WIND database	Dollar/barrel
Carbon price drivers in the domestic market	Qinhuangdao port thermal coal (Q5500) market price	Coal	Steel house	Yuan/ton
	The average temperature in Shenzhen	Temp	WIND database	Centigrade
	The spot price of Pacific Rim crude oil (China’s victory)	Crude	WIND database	Dollar/barrel
	CSI industrial index	Indus	Sina Finance	Spot
	Shenwan 300 index	Index	Shanghai Shenyin Wanguo Securities Institute	Spot
	Shenzhen air quality comprehensive index	Air	Ministry of Environmental Protection	/

**Table 3:** Variable descriptive statistics.

Variables	Mean	Max	Min	Std. dev	Skew	Kurt	N
Carbon	31.8899	88.45	3.12	17.5178	1.0119	4.1205	1406
CER	0.2774	0.68	0.01	0.1293	0.42155	3.2271	1406
Coal	541.1145	1042.5	370.00	94.4871	0.3661	5.6631	1406
Crude	57.4297	110.36	19.7	20.4047	0.8852	3.4112	1406
Air	54.867	180	19	23.401	1.3193	6.61946	1406
ARA-coal	68.035	104.13	37.00	16.644	0.3595	1.9690	1406
Brent	62.1339	115.3	14.75	20.3564	0.9251	3.4302	1406
Index	2630.403	4238.02	1624.02	564.4532	0.4427	3.0698	1406
Indus	3089.496	6591.508	1935.967	778.2419	1.5037	6.6918	1406
Temp	0.3514	1	0	0.477663	0.622371	1.387345	1406

Std. dev., the standard deviation; Skew., the skewness; Kurt., the kurtosis; and N, the number of observations.

**Table 4:** Johansen co-integration test.

Hypothesized No. of CE(*s*)	Eigenvalue	Trace	Max-Eigen
R ≤ 0	0.267993	567.9001 (≤0.001)	302.9183 (0.0001)
R ≤ 1	0.091375	264.9818 (≤0.001)	93.04386 (≤0.001)
R ≤ 2	0.057895	171.9379 (0.0088)	57.90894 (0.0123)
R ≤ 3	0.04171	114.029 (0.2042)	41.36939 (0.1515)

Note: () probability value; denote a test statistic is statistically significant at the 1%, 5% or 10% level of significance.

**Table 5:** Granger causality test.

Null Hypothesis	F-Statistic	Prob.	Results
Carbon does not Granger cause Brent	3.11621	0.0448	Refused
Brent does not Granger cause carbon	5.7497	0.0033	refused
Crude does not Granger cause carbon	6.26317	0.002	Refused
Carbon does not Granger cause crude	2.8023	0.0612	refused
Temp does not Granger cause carbon	0.25081	0.7782	Accepted
Carbon does not Granger Cause Temp	3.62069	0.0271	Refused
Coal does not Granger Cause Carbon	0.37359	0.6884	Accepted
Carbon does not Granger Cause Coal	2.85507	0.6884	Accepted
The index does not Granger Cause Carbon	2.40945	0.0904	Refused
Carbon does not Granger Cause Index	2.47555	0.0846	Refused

**Table 6:** Correlation test results.

Variables	Carbon	CER	Coal	Crude	Air	ARA-Coal	Brent	Index	Indus
CER	−0.05237	1	-----	-----	-----	-----	-----	-----	-----
	(0.102)								
Coal	0.173385	−0.4201	1	-----	-----	-----	-----	-----	-----
	(≤0.001 ***)	(≤0.001 ***)	-----						
Crude	0.814118	−0.33387	0.38771	1	-----	-----	-----	-----	-----
	(≤0.001 ***)	(≤0.001 ***)	(≤0.001 ***)	-----					
Air	0.000665	0.014621	0.01044	0.00396	1	-----	-----	-----	-----
	(0.9834)	(0.6482)	(0.7447)	(0.9017)	-----				
ARA-coal	0.330979	−0.344	0.87298	0.59407	0.00876	1	-----	-----	-----
	(≤0.001 ***)	(≤0.001 ***)	(≤0.001 ***)	(≤0.001 ***)	(0.7846)	-----			
Brent	0.824797	−0.30656	0.35446	0.99462	−0.001385	0.56085	1	-----	-----
	(≤0.001 ***)	(≤0.001 ***)	(≤0.001 ***)	(≤0.001 ***)	(0.9655)	(≤0.001 ***)	-----		
Index	−0.61573	0.187584	−0.28428	−0.6188	−0.00911	−0.36215	−0.6419	1	-----
	(≤0.001 ***)	(≤0.001 ***)	(≤0.001 ***)	(≤0.001 ***)	(0.7762)	(≤0.001 ***)	(≤0.001 ***)	-----	
Indus	−0.56501	0.262155	−0.36355	−0.5782	−0.008599	−0.40212	−0.5992	0.96962	1
	(≤0.001 ***)	(≤0.001 ***)	(≤0.001 ***)	(≤0.001 ***)	(0.7885)	(≤0.001 ***)	(≤0.001 ***)	(≤0.001 ***)	-----
Temp	−0.2136	−0.21293	−0.26105	−0.2107	0.021213	−0.32769	−0.1964	0.00115	−0.0633
	(≤0.001 ***)	(≤0.001 ***)	(≤0.001 ***)	(≤0.001 ***)	(0.508)	(≤0.001 ***)	(≤0.001 ***)	(0.9715)	(0.048 **)

**, *** denote a test statistic is statistically significant at the 5%, or 10% level of significance.

**Table 7:** OLS regression results.

Variables	Non-Standardized Coefficient	t-Statistic	*p*	VIF
CER	26.25082	11.7337	≤0.001	1.711165
Coal	0.026536	3.245864	0.0012	5.963452
Crude	0.70063	5.931098	≤0.001	134.3242
Air	−0.111718	−0.342546	0.732	1.006403
ARA-coal	−0.488859	−8.960124	≤0.001	6.840569
Brent	−0.081242	−0.671511	0.5021	130.0989
Index	0.005298	1.857073	0.0636	24.8853
Indus	−0.006457	−4.241722	≤0.001	24.99086
Temp	−2.675742	−3.671472	0.3	1.515843
C	27.81306	5.989027	≤0.001	-----
R^2^	0.968842	------	-----	-----
F-statistics	356.9948	------	-----	-----
*p*	≤0.001	------	-----	-----

**Table 8:** Ridge regression estimators of variables (k = 0.1).

Variables	Non-Standardized Coefficient	t-Statistic	*p*	VIF
Coal	−0.00115989	3.173	0.00151 ***	1.567
Temp	−2.6645335	3.719	0.2	1.246
Crude	−0.26584276	8.208	≤0.001 ***	1.078
Indus	0.00235551	4.223	≤0.001 ***	0.621
Index	0.00304198	1.524	0.12756	0.873
Air	−0.03278454	0.233	≤0.001***	1.963
CER	17.90157308	11.489	≤0.001 ***	2.158
ARA-coal	−0.20011181	8.728	≤0.001 ***	0.9654
Brent	−0.2618561	1.922	0.05466 *	0.8765
C	34.16795176	3.362	0.023 **	-----
R^2^	0.869686414	-----	-----	-----
F-statistics	333.1973346	-----	-----	-----
*p*	≤0.001	-----	-----	-----

*, **, *** denote a test statistic is statistically significant at the 1%, 5%, or 10% level of significance; VIF indicates expansion factor.

## Data Availability

The data of this study can be obtained from the corresponding author upon reasonable request.
